# A variable- and person-centered study of physical exercise volume and psychological flourishing among Chinese college students: serial indirect associations of self-compassion and body appreciation and latent profile analysis

**DOI:** 10.3389/fpsyg.2026.1880717

**Published:** 2026-07-07

**Authors:** Qiuxia Wan, Tian Gao, Fang Wang, Qianxiao Zhang

**Affiliations:** 1 School of Sports Training, Chengdu Sport University, Chengdu, China; 2 Faculty of Education, Shinawatra University, Sam Khok, Thailand; 3 College of Teacher Education, Batangas State University, Batangas, Philippines; 4 College of Sports and Great Health, Sichuan Technology and Business University, Sichuan, China

**Keywords:** body appreciation, Chinese college students, latent profile analysis, physical exercise volume, psychological flourishing, self-compassion, serial indirect associations

## Abstract

**Introduction:**

Promoting psychological flourishing among college students is a central goal of positive mental health research. Guided by an embodied positive psychology perspective, this cross-sectional study examined the association between physical exercise volume and psychological flourishing among Chinese college students, focusing on indirect associations through self-compassion and body appreciation and on heterogeneity in positive self-body relationship profiles.

**Methods:**

A total of 2,401 students from 15 universities in Sichuan Province, China, completed measures of physical exercise volume, self-compassion, body appreciation, and psychological flourishing. Structural equation modeling was used to examine direct, specific indirect, and serial indirect associations. Latent profile analysis was used to identify configurations of self-compassion dimensions and body appreciation, and R3STEP and BCH analyses were used to examine associations with physical exercise volume and differences in psychological flourishing.

**Results:**

Physical exercise volume was positively associated with psychological flourishing. Significant indirect associations were observed via self-compassion, body appreciation, and the serial association through self-compassion and body appreciation. The indirect association via self-compassion was larger than that via body appreciation. Four profiles were identified: low self-compassion-low body appreciation, high self-compassion-high body appreciation, mindfulness-dominant moderate, and low-mindfulness moderate. Higher physical exercise volume was associated with lower odds of the low self-compassion-low body appreciation profile and higher odds of the high self-compassion-high body appreciation profile relative to the low-mindfulness moderate profile. Psychological flourishing was highest in the high self-compassion-high body appreciation profile, lowest in the low self-compassion-low body appreciation profile, and intermediate and statistically comparable in the two moderate profiles.

**Conclusion:**

Integrating average-level indirect associations with person-centered self-body configurations provides a more nuanced account of the relationship between physical exercise and psychological flourishing among college students. Given the cross-sectional design, the findings should be interpreted as theoretically meaningful associations rather than causal mechanisms.

## 1 Introduction

### 1.1 Physical exercise volume and psychological flourishing

Promoting college students' mental health has increasingly moved beyond the prevention of psychological symptoms toward the cultivation of optimal functioning (Shdaifat et al., 2024). In this positive mental health perspective, flourishing refers not merely to feeling good, but also to functioning effectively in important domains of life, including purpose, competence, optimism, self-respect, positive relationships, and contribution to others (Keyes, 2002; Huppert and So, 2013; Diener et al., 2010). This perspective is particularly relevant for college students, who are navigating academic demands, identity development, interpersonal transitions, and future career uncertainty. For this population, psychological flourishing represents a desirable developmental outcome: students who flourish are not only less distressed, but also more capable of engaging meaningfully with study, relationships, and personal growth.

Physical exercise is one of the most accessible health-related behaviors associated with positive student development. The World Health Organization emphasizes that physical activity can be characterized by its frequency, intensity, and duration, and that regular activity provides broad health benefits across populations (World Health Organization, 2020). For college students, physical exercise is not simply a matter of energy expenditure or physical fitness; it is also a repeated context in which individuals experience bodily competence, effort regulation, mastery, vitality, and social connectedness. These experiences may be closely connected with the psychological resources that underlie flourishing (Li K. et al., 2025; Zhang et al., 2026). Empirical evidence among Chinese youths and undergraduates has also shown that physical activity is positively associated with flourishing, and that this association may involve positive psychological resources such as meaning in life and self-efficacy (Zhou and Huo, 2022).

However, existing research has paid relatively limited attention to how physical exercise volume may be associated with flourishing through self-related and body-related psychological processes. Many studies have focused on direct associations between exercise and wellbeing, or on general psychological correlates such as self-efficacy, meaning, and resilience. Less attention has been given to the possibility that exercise is psychologically meaningful because it is inherently embodied: it is performed through the body, evaluated through bodily experience, and often interpreted in relation to self-worth, discipline, comparison, and body image. From this perspective, exercise may be linked to flourishing not only because students move more, but also because movement provides repeated opportunities to interpret bodily feedback, regulate effort, experience capability, and relate to the body in more or less appreciative ways. Therefore, the present study focuses on two theoretically relevant self–body resources—self-compassion and body appreciation—to examine the association between physical exercise volume and psychological flourishing among Chinese college students.

H1. Physical exercise volume is positively associated with psychological flourishing among Chinese college students.

### 1.2 The indirect role of self-compassion

Self-compassion provides a theoretically meaningful bridge between physical exercise and psychological flourishing (Hall et al., 2025; Zhang et al., 2025). Neff (2003) conceptualized self-compassion as a healthy way of relating to oneself in moments of difficulty, involving self-kindness rather than self-judgment, common humanity rather than isolation, and mindfulness rather than over-identification. In the context of college life, self-compassion may help students respond to academic pressure, interpersonal setbacks, and self-doubt with greater emotional balance and less harsh self-criticism (Huang et al., 2025). The Chinese version of the Self-Compassion Scale has shown acceptable psychometric properties among Chinese undergraduate students, supporting its applicability in the population targeted by the present study (Chen et al., 2011).

The relevance of self-compassion to physical exercise can be understood from a self-regulation perspective. Self-regulation theories emphasize that individuals monitor feedback, compare current states with goals or standards, and adjust behavior and affect in response to discrepancies (Carver and Scheier, 1982). Exercise contexts make such self-regulatory processes especially salient. Students repeatedly encounter effort, fatigue, bodily limitations, performance fluctuations, social comparison, and irregular progress. These experiences require regulation not only at the behavioral level, such as adjusting intensity or maintaining participation, but also at the self-evaluative level, such as interpreting temporary difficulty without excessive self-blame. Self-compassion may be particularly relevant in this context because it provides a kind, balanced, and non-isolating way of responding to imperfection and challenge.

Physical exercise volume may therefore be associated with higher self-compassion for several reasons. First, regular exercise often requires individuals to listen to their bodies, regulate effort, tolerate temporary discomfort, and recover from setbacks, all of which may be accompanied by a more patient and caring relationship with the self (Zhu and Wen, 2025). Second, exercise-related mastery experiences may be linked to lower self-defeating appraisals and a more constructive self-orientation. Third, some forms of exercise, especially those emphasizing body awareness and non-judgmental attention, may be especially compatible with self-compassionate responses to physical and psychological challenges (Wong et al., 2026). A systematic review and meta-analysis found a significant relationship between physical activity and self-compassion, indicating that individuals who engage in more physical activity tend to report higher self-compassion (Wong et al., 2021). Recent evidence among college students also indicates that self-compassion is closely involved in the association between body-related social experiences and physical activity, suggesting that self-compassion is a relevant self-regulatory resource in physical-activity contexts marked by appearance comparison and body-related evaluation (Li X. et al., 2025).

Self-compassion is also closely tied to flourishing. Because flourishing involves meaningful functioning rather than the mere absence of distress, it depends on the ability to maintain self-acceptance, emotional balance, and adaptive engagement when facing difficulty (Liu et al., 2024). A meta-analysis showed that self-compassion is positively associated with multiple forms of wellbeing and is especially strongly related to psychological wellbeing (Zessin et al., 2015). Thus, students with greater self-compassion may be more likely to experience purpose, competence, optimism, and positive self-regard—core components of psychological flourishing.

H2. The association between physical exercise volume and psychological flourishing is expected to include a significant indirect association via self-compassion.

### 1.3 The indirect role of body appreciation

Body appreciation represents another important self–body resource in the association between physical exercise and psychological flourishing (Linardon et al., 2022). Unlike traditional body image research that focuses on dissatisfaction, shame, or appearance concerns, body appreciation reflects a positive orientation toward the body, including acceptance, respect, care, and gratitude for the body's functions (Tylka and Wood-Barcalow, 2015a). Positive body image is not simply the absence of negative body image; rather, it involves an active and respectful relationship with the body as a source of agency, experience, and participation in life (Tylka and Wood-Barcalow, 2015b; Piran, 2019). The Body Appreciation Scale-2 has been widely used to assess positive body image, and its Mandarin Chinese version has been validated among college students from the Chinese mainland, with evidence supporting its one-factor structure, construct validity, incremental validity, and measurement invariance across gender (Ma et al., 2022).

Physical exercise may be associated with body appreciation because it can shift students' attention from how the body looks to what the body can do. Through movement, students may experience strength, endurance, coordination, flexibility, vitality, rhythm, recovery, and bodily competence. These experiences can help the body become a source of capability rather than merely an object of appearance evaluation. Research on body functionality has similarly emphasized that attending to the body's functions can support more positive body image and reduce self-objectification (Alleva et al., 2015). In this sense, exercise is relevant to positive body image because the body is appreciated not only for meeting external appearance standards, but for enabling action, growth, connection, and engagement with life. Intervention evidence among female college students further suggests that a basketball-based exercise program can enhance positive body image through embodied experience and self-compassion, supporting the relevance of exercise-related embodiment and self-related processes in positive body image development (Zhu and Wen, 2025). Recent longitudinal evidence among college students also suggests that physical activity is meaningfully related to body appreciation, further supporting the relevance of body appreciation in exercise-related wellbeing research (Zhou et al., 2024).

Body appreciation may, in turn, be positively associated with psychological flourishing. Students who respect and value their bodies may be less consumed by appearance-based self-criticism and more able to invest psychological energy in meaningful goals, relationships, and self-development. Body appreciation may be particularly relevant to flourishing because it reflects a positive and respectful relationship with the body as the basis of action, agency, vitality, and engagement with life. A systematic review and meta-analysis found that body appreciation is positively associated with adaptive wellbeing constructs, and that these associations are not simply reducible to the absence of negative body image (Linardon et al., 2022). This suggests that body appreciation is not merely a protective factor against body dissatisfaction, but a positive psychological resource in its own right.

H3. The association between physical exercise volume and psychological flourishing is expected to include a significant indirect association via body appreciation.

### 1.4 Serial indirect associations involving self-compassion and body appreciation

Although self-compassion and body appreciation may each be involved in the association between physical exercise volume and psychological flourishing, their relationship may also be meaningfully organized in sequence. The present study proposes that self-compassion is positioned prior to body appreciation in the statistical model. This ordering is theoretically grounded in the distinction between a broad self-relational orientation and a more body-specific positive attitude. Self-compassion refers to how individuals relate to themselves in the face of difficulty, inadequacy, and imperfection, whereas body appreciation refers to how individuals accept, respect, and value their bodies. Thus, self-compassion may provide a broader interpretive frame within which bodily experiences are evaluated less harshly and more appreciatively.

This ordering is especially relevant in exercise contexts. Physical exercise provides repeated embodied situations in which students encounter bodily feedback, effort, fatigue, comparison, temporary failure, and performance variability. Such experiences do not have a fixed psychological meaning; rather, they are interpreted in relation to the self. A student who experiences fatigue during exercise may interpret it as personal failure, lack of discipline, or bodily inadequacy. Another student may interpret the same experience as a normal part of effort, learning, and bodily adaptation. Self-compassion may support the latter interpretation by allowing students to respond to difficulty with self-kindness, common humanity, and balanced awareness rather than harsh self-judgment. This broader self-compassionate orientation may then provide a psychological basis for body appreciation, because students who relate to themselves with less criticism may be more likely to value the body for its functionality, resilience, and lived experience.

This sequence is also consistent with empirical evidence. Cox et al. (2019) found that self-compassion was positively related to body appreciation and that body appreciation mediated the relationship between self-compassion and intrinsic motivation for physical activity in college women. Their longitudinal findings further showed that changes in self-compassion were prospectively associated with changes in body appreciation, suggesting that a more compassionate self-relation may create psychological conditions for a more appreciative body relation. These findings are directly relevant to the present study because they position self-compassion as a broader self-regulatory and self-accepting resource that can support positive body image.

At the same time, the proposed ordering should not be interpreted as excluding other theoretically plausible directions. Body appreciation may also support self-compassion by reducing body-related shame, appearance-based self-criticism, and alienation from the body. A more appreciative body relation may make it easier for students to respond to themselves with kindness and less judgment. Therefore, the proposed sequence is best understood as a theory-guided model of statistical indirect associations rather than evidence of temporal or causal ordering.

These self–body processes may be particularly relevant in the Chinese college context. Cross-cultural theories suggest that many Asian cultural contexts place greater emphasis on relational and interdependent self-construals, in which self-evaluation is often shaped by social expectations and the perceived reactions of others (Markus and Kitayama, 1991). Related cross-cultural work has also suggested that self-critical forms of evaluation may be more salient in some East Asian contexts than in cultural contexts that strongly emphasize positive self-regard (Heine et al., 1999). For Chinese college students, academic competition, peer comparison, collective expectations, and appearance-related social pressures may make self-evaluation and body evaluation especially meaningful in everyday life. Recent evidence also indicates that body-related social networking experiences and upward appearance comparisons are relevant to college students' physical activity, with self-compassion playing a role in this association (Li X. et al., 2025). Under such conditions, physical exercise volume may be connected with flourishing not only through participation itself, but also through how students interpret exercise-related bodily experiences in relation to the self and the body. Self-compassion may help students respond to effort and imperfection with less harshness, while body appreciation may help them value the body beyond appearance-based standards.

Accordingly, the present study examines a serial indirect association model in which physical exercise volume is associated with psychological flourishing via self-compassion and body appreciation in sequence.

H4. The association between physical exercise volume and psychological flourishing is expected to include a significant serial indirect association via self-compassion and body appreciation. Specifically, physical exercise volume is positively associated with self-compassion, which is positively associated with body appreciation, which in turn is positively associated with psychological flourishing.

### 1.5 Latent profile analysis of positive self–body relationship

Although the serial indirect association model can clarify average associations among physical exercise volume, self-compassion, body appreciation, and psychological flourishing, it cannot fully capture heterogeneity among students. Variable-centered approaches assume that the same structural relations apply to the sample as a whole (Ren et al., 2025). However, students may show different configurations of self-compassion and body appreciation. For example, some students may report consistently high self-compassion and high body appreciation, whereas others may show moderate self-compassion but low body appreciation, or high body appreciation alongside relatively strong self-judgment (Miao et al., 2025). These patterns are theoretically meaningful because self-compassion and body appreciation are related but not identical constructs.

Latent profile analysis offers a person-centered approach for identifying unobserved subgroups of individuals who share similar patterns across continuous indicators. This method is particularly useful when researchers expect that psychological resources may combine in different ways across individuals rather than operate uniformly across a population (Nylund-Gibson and Choi, 2018; Spurk et al., 2020; Freire et al., 2020). In the present study, latent profiles will be identified using the six dimensions of self-compassion and body appreciation, representing different profiles of students' positive self–body relationship. Because the dimensions of self-compassion may not always co-occur at the same level within individuals, a person-centered approach can reveal whether students show balanced or uneven configurations of self-kindness, mindfulness, reduced self-judgment, common humanity, and body appreciation. This person-centered analysis complements the serial indirect association model by addressing not only how variables are associated on average, but also how self-related and body-related resources are configured within individuals.

The LPA component also extends the practical implications of the study. If distinct positive self–body relationship profiles are identified, universities may be able to design more targeted physical exercise and mental health programs. For example, students with low self-compassion and low body appreciation may require interventions emphasizing self-kindness and body acceptance, whereas students with adequate self-compassion but low body appreciation may benefit more from exercise programs that highlight body functionality, bodily competence, and non-appearance-based body value. Furthermore, examining whether physical exercise volume is associated with profile membership and whether profiles differ in psychological flourishing can clarify how exercise behavior, positive self–body configurations, and flourishing are connected at the person level.

RQ1. What latent profiles of positive self–body relationship can be identified among Chinese college students based on self-compassion and body appreciation?

RQ2. Is physical exercise volume associated with membership in different latent profiles of positive self–body relationship?

RQ3. Do students in different latent profiles differ in psychological flourishing?

### 1.6 The present study

Taken together, the present study integrates variable-centered and person-centered perspectives to examine the association between physical exercise volume and psychological flourishing among Chinese college students. The variable-centered component examines a theory-guided serial indirect association model in which self-compassion and body appreciation are sequentially involved in the association between physical exercise volume and psychological flourishing. The person-centered component uses latent profile analysis to identify distinct positive self–body relationship profiles based on self-compassion and body appreciation, and further examines whether physical exercise volume is associated with profile membership and whether psychological flourishing differs across profiles.

**Figure 1:**
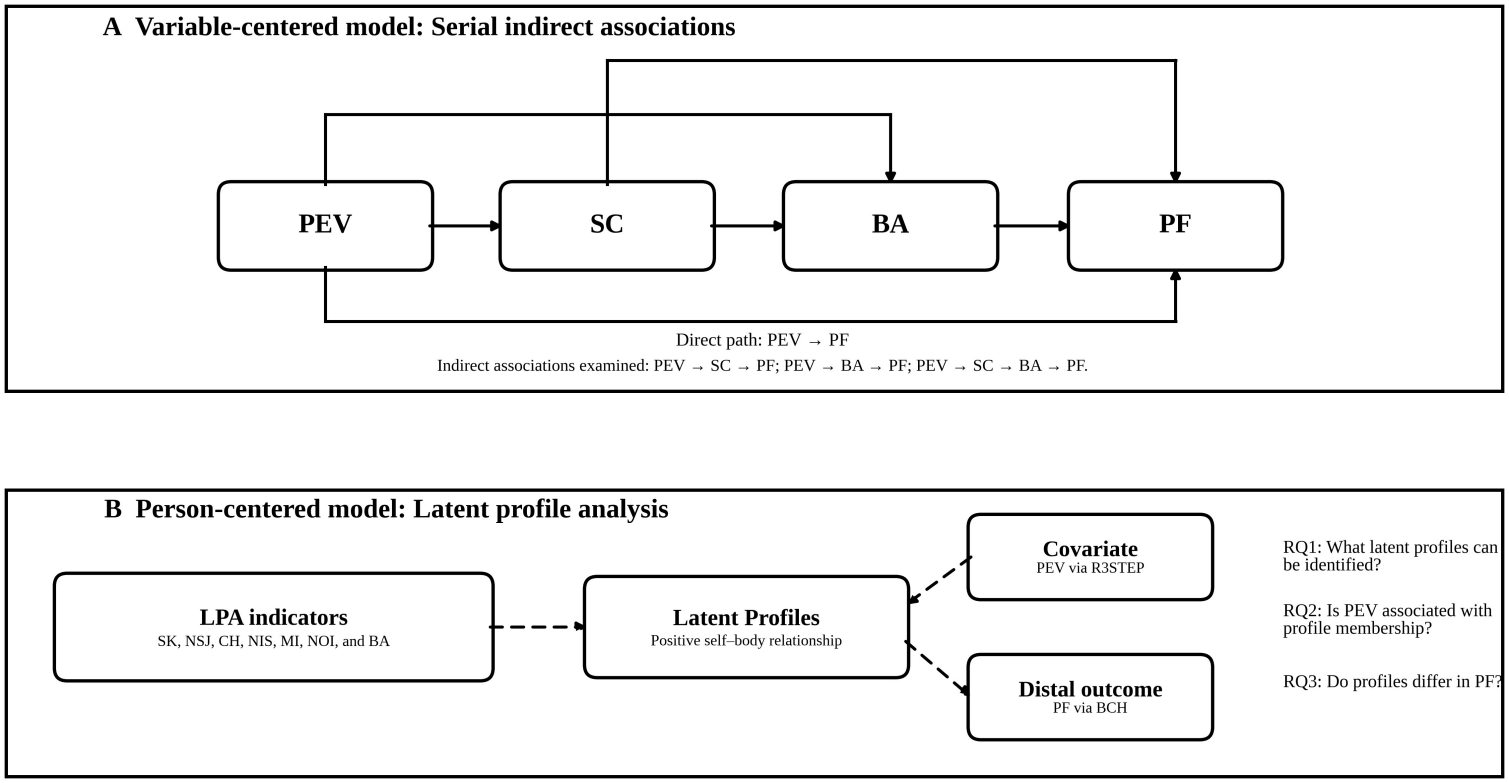
Conceptual model of the serial indirect association and latent profile analysis framework. **(A)** Variable-centered model: serial indirect associations. **(B)** Person-centered model: latent profile analysis.

This study contributes to the literature in three ways. First, it shifts attention from symptom reduction to flourishing, thereby aligning college student mental health research with a positive mental health framework. Second, it examines the exercise–flourishing association from an embodied psychological perspective, highlighting how physical exercise volume may be linked to flourishing through students' relationships with themselves and their bodies. Third, it combines a variable-centered model of indirect associations with latent profile analysis, allowing the study to examine both average-level associations and individual heterogeneity. Given the cross-sectional design, the proposed model is interpreted as a theoretically specified pattern of associations rather than as evidence of causal or temporal ordering. The hypothesized conceptual model is presented in Figure 1.

## 2 Methods

### 2.1 Participants and procedure

Data were collected through Wenjuanxing, a widely used online survey platform in China, between October 15 and November 15, 2025. Participants were recruited from 15 universities in Sichuan Province, China, including Sichuan University, Southwestern University of Finance and Economics, Southwest Jiaotong University, University of Electronic Science and Technology of China, and other participating institutions. The survey link was distributed through university-affiliated online channels, including class groups, student organizations, and campus social media networks. Before completing the questionnaire, participants were informed of the study purpose, the anonymous nature of their responses, the voluntary nature of participation, and their right to withdraw from the study at any time without penalty. Online informed consent was obtained from all participants prior to their participation. The study protocol was reviewed and approved by the Ethics Committee of the School of Sports Training, Chengdu Sport University (Approval No. CTYLL2024022).

A total of 2,571 questionnaires were initially collected. Because all survey items were set as mandatory on Wenjuanxing, no responses were excluded due to missing values. Following a pre-specified data-screening procedure, 170 invalid responses were removed, including questionnaires completed in less than 60 s (*n* = 81), patterned or straight-line responses across scale items (*n* = 52), logically inconsistent responses or failed quality-control checks (*n* = 21), and duplicate or highly similar submissions identified by IP address, device information, submission time, and response pattern (*n* = 16). The final valid sample consisted of 2,401 college students, yielding a valid response rate of 93.39%.

The demographic characteristics of the valid sample are presented in Table 1. Participants were predominantly female (54.94%), and most were between 19 and 21 years old. The sample covered students from different grades, academic disciplines, residence backgrounds, and university types, indicating adequate heterogeneity for describing the sample characteristics and interpreting the general context of the findings.

**Table 1:** Demographic characteristics of the valid sample (*N* = 2,401).

Variable	Category	*n*	%
Gender	Male	1,082	45.06
	Female	1,319	54.94
Age	≤ 18 years	335	13.95
	19 years	542	22.57
	20 years	610	25.41
	21 years	516	21.49
	22 years	281	11.70
	≥23 years	117	4.87
Grade	Freshman	609	25.36
	Sophomore	684	28.49
	Junior	623	25.95
	Senior or above	485	20.20
Academic discipline	Science and engineering	744	30.99
	Economics and management	487	20.28
	Humanities and social sciences	512	21.32
	Medicine and health sciences	231	9.62
	Education, arts, and sports	296	12.33
	Agriculture and other majors	131	5.46
Residence background	Urban	1,176	48.98
	Rural	1,225	51.02
Only-child status	Yes	801	33.36
	No	1,600	66.64
Ethnicity	Han ethnicity	2,244	93.46
	Ethnic minority groups	157	6.54
University type	Comprehensive universities	578	24.07
	Science and engineering universities	652	27.16
	Finance/economics universities	342	14.24
	Normal universities	314	13.08
	Medical, agricultural, or other universities	515	21.45

### 2.2 Measurements

All variables were measured using self-report questionnaires that have been widely used in previous psychological and physical activity research. To maintain response-format consistency across the survey and reduce response-scale switching during online completion, the psychological scales were administered using a five-point Likert-type format, with higher scores indicating higher levels of the corresponding construct. For scales originally developed in English, validated Chinese versions were used where available, and the psychometric properties of the translated instruments have been supported in Chinese samples. Because the Flourishing Scale was originally developed with a seven-point response format, the use of a five-point format in the present study was treated as a response-format adaptation. Accordingly, the reliability and factorial validity of the adapted five-point FS were specifically examined in the present sample.

#### 2.2.1 Physical exercise volume

Physical activity was assessed using the Physical Activity Rating Scale-3 (PARS-3), which was revised by Liang (1994) and has been widely used in Chinese research on physical exercise among students. The scale measures exercise volume over the past month using three indicators: exercise intensity, exercise duration, and exercise frequency. Each indicator is assessed with five ordered response categories. A representative item is, “How intense is your physical exercise?” Following the standard scoring method, intensity and frequency are coded from 1 to 5, while duration is recoded from 0 to 4. The total exercise volume score is calculated as intensity × duration × frequency, with higher scores indicating a greater level of physical activity. The PARS-3 is an index-type measure rather than a reflective psychological scale; therefore, internal consistency reliability is not estimated for this measure. Previous Chinese sources have reported the PARS-3 as a commonly used measure of physical exercise volume, with a test–retest reliability of 0.82. Because PARS-3 uses a multiplicative scoring method and the duration component is recoded from 0 to 4, participants reporting no exercise duration may obtain a total score of 0. Therefore, the distribution of physical exercise volume was inspected in the preliminary analyses, including range, median, interquartile range, skewness, kurtosis, and the proportion of zero scores, to evaluate whether floor concentration was present.

#### 2.2.2 Self-compassion

Self-compassion was measured using the Chinese version of the Self-Compassion Scale (SCS; Neff, 2003; Chen et al., 2011). The SCS contains 26 items and assesses six dimensions of self-compassion: self-kindness, self-judgment, common humanity, isolation, mindfulness, and over-identification. A representative item is, “When I'm going through a very hard time, I give myself the caring and tenderness I need.” Participants rated each item on a five-point scale ranging from 1 = almost never to 5 = almost always. According to the scoring instructions, items from the negative dimensions of self-judgment, isolation, and over-identification are reverse-scored before calculating the total self-compassion score. Higher scores indicate a higher level of self-compassion. The Chinese version was validated among Chinese undergraduate students, and exploratory and confirmatory factor analyses supported the six-factor structure. Chen et al. (2011) also reported good internal consistency and test–retest reliability for the Chinese version of the scale.

#### 2.2.3 Body appreciation

Body appreciation was assessed using the Mandarin Chinese version of the Body Appreciation Scale-2 (BAS-2; Tylka and Wood-Barcalow, 2015a; Ma et al., 2022). The BAS-2 consists of 10 items and measures individuals' acceptance of, respect for, and positive attitudes toward their bodies. A representative item is, “I respect my body.” Participants responded on a five-point scale ranging from 1 = never to 5 = always. The mean score of the 10 items was calculated, with higher scores indicating greater body appreciation. The BAS-2 was originally developed as a unidimensional measure, and the Mandarin Chinese version has been validated among college students from the Chinese mainland. Ma et al. (2022) found support for its one-factor structure, internal consistency, test–retest reliability, convergent validity, discriminant validity, criterion-related validity, and incremental validity.

#### 2.2.4 Psychological flourishing

Psychological flourishing was measured using the Flourishing Scale (FS; Diener et al., 2010). The FS contains eight items and assesses perceived success in important domains of psychological functioning, such as meaning, competence, optimism, social relationships, and contribution to others. A representative item is, “I lead a purposeful and meaningful life.” In the present study, participants responded using a five-point agreement scale ranging from 1 = strongly disagree to 5 = strongly agree. The mean score of the eight items was calculated, with higher scores indicating a higher level of psychological flourishing. The original FS was developed as a brief unidimensional measure of psychological wellbeing, and the simplified Chinese version has been psychometrically evaluated in Chinese samples (Tang et al., 2016). Previous Chinese validation studies have supported the applicability of the FS in Chinese contexts, including evidence for model fit, reliability, and measurement stability. The five-point administration of the FS was adopted to maintain response-format consistency across the psychological measures, reduce response burden in the online survey, and minimize potential confusion caused by switching between different response scales. Because changing the response format may affect response variability, scale sensitivity, and comparability with studies using the original seven-point version, the adapted FS was evaluated separately in the present sample. The adapted five-point FS showed excellent internal consistency, McDonald's ω = 0.933. A single-factor CFA of the eight FS items supported the unidimensional structure of the adapted scale, χ^2^/df = 3.478, CFI = 0.995, TLI = 0.994, SRMR = 0.011, RMSEA = 0.032. Standardized factor loadings ranged from 0.733 to 0.816.

### 2.3 Data analysis

Data analyses were conducted using IBM SPSS Statistics 29.0, IBM SPSS Amos 26.0, and Mplus 8.3. SPSS 29.0 was used for data screening, descriptive statistics, reliability analyses, Harman's single-factor test, full collinearity VIF assessment, and Pearson correlation analyses. Amos 26.0 was used to conduct confirmatory factor analysis (CFA), compare alternative measurement models, evaluate the adapted FS separately, and test the hypothesized structural equation model and indirect associations. Mplus 8.3 was used to conduct latent profile analysis (LPA), the R3STEP multinomial logistic regression, and the BCH auxiliary outcome analysis. All statistical tests were two-tailed, and statistical significance was evaluated at *p* < 0.05.

Demographic variables were summarized descriptively to characterize the sample rather than entered as covariates in the focal models. This decision was made for several reasons. First, the primary purpose of the study was to examine theoretically specified associations among physical exercise volume, self-compassion, body appreciation, psychological flourishing, and positive self–body relationship profiles, rather than to estimate demographic-adjusted group differences. Second, demographic variables such as gender, grade, academic discipline, residence background, and university type were not specified as theoretical confounders in the hypothesized model. Third, the automatic inclusion of demographic variables as statistical controls may introduce difficult-to-interpret adjustments when their theoretical roles are unclear (Spector and Brannick, 2011). Fourth, the person-centered component of the study aimed to identify naturally occurring configurations of self-compassion dimensions and body appreciation; therefore, the main LPA, R3STEP, and BCH analyses were kept aligned with the focal research questions. Accordingly, no additional demographic-adjusted sensitivity model was specified. The potential relevance of demographic heterogeneity is considered in the interpretation of the sample characteristics and in the limitations and future directions.

First, common method bias was examined because all focal variables were collected through self-report questionnaires at a single time point. Procedural remedies were implemented during data collection, including anonymous responding, voluntary participation, and the clarification that there were no right or wrong answers. Statistically, Harman's single-factor test was conducted in SPSS by entering all item-level indicators of the reflective psychological measures and the three original indicators of physical exercise volume into an unrotated exploratory factor analysis. Common method bias was considered unlikely to be severe when no single factor accounted for the majority of the total variance. In addition, a series of competing CFA models was estimated in Amos to compare the hypothesized multi-factor measurement model with alternative models in which theoretically distinct constructs were combined. This approach follows the recommendation that common method bias should not be assessed solely through a single-factor test, but should also be evaluated using theory-based measurement model comparisons (Podsakoff et al., 2003).

To further evaluate potential common method bias, full collinearity VIFs were also calculated for the focal construct scores. In this procedure, each focal construct score was regressed on the remaining focal construct scores, and the resulting variance inflation factors were inspected. VIF values at or below 3.3 were interpreted as not indicating serious common-method-related collinearity (Kock, 2015). This additional diagnostic was used as a complementary procedure rather than as definitive evidence that common method bias was absent.

Second, preliminary analyses were conducted to examine descriptive statistics, reliability, convergent validity, discriminant validity, and bivariate associations among the study variables. Means, standard deviations, skewness, kurtosis, and Pearson correlation coefficients were calculated in SPSS. Because physical exercise volume was calculated using the multiplicative PARS-3 scoring method, its distribution was further inspected for potential floor concentration. Specifically, the range, minimum, maximum, median, interquartile range, skewness, kurtosis, and the number and percentage of zero scores were reported. For the psychological scales, internal consistency reliability was evaluated using McDonald's omega. Values of 0.70 or above were considered acceptable for research purposes (McDonald, 1999; Nunnally and Bernstein, 1994). Because physical exercise volume was calculated as an index score based on exercise intensity, duration, and frequency, it was not treated as a reflective latent construct and was therefore not evaluated using internal consistency reliability, composite reliability, or average variance extracted.

Third, CFA was conducted in Amos to evaluate the measurement structure of the reflective constructs, including the six self-compassion dimensions, body appreciation, and psychological flourishing. A separate one-factor CFA was also conducted for the adapted five-point FS to examine whether the modified response format retained the expected unidimensional structure. Physical exercise volume was not included in the CFA model because it was computed as an observed index score rather than modeled as a reflective factor. Model fit was evaluated using multiple indices, including the chi-square statistic divided by degrees of freedom (χ^2^/df), comparative fit index (CFI), Tucker–Lewis index (TLI), standardized root mean square residual (SRMR), and root mean square error of approximation (RMSEA). Because the chi-square statistic is sensitive to large sample sizes, model fit was judged primarily using approximate fit indices. CFI and TLI values of 0.90 or above were considered acceptable and values of 0.95 or above were considered good. SRMR and RMSEA values below 0.08 were considered acceptable, and values close to or below 0.06 were interpreted as indicating good fit (Hu and Bentler, 1999; Kline, 2023). Convergent validity was evaluated using standardized factor loadings, composite reliability (CR), and average variance extracted (AVE). Standardized factor loadings of 0.50 or above, CR values of 0.70 or above, and AVE values of 0.50 or above were considered evidence of acceptable convergent validity (Fornell and Larcker, 1981; Hair et al., 2019). Discriminant validity was assessed using the Fornell–Larcker criterion and the heterotrait–monotrait ratio of correlations (HTMT). Discriminant validity was considered supported when the square root of each construct's AVE exceeded its correlations with other constructs, and when HTMT values were below the conservative threshold of 0.85 (Fornell and Larcker, 1981; Henseler et al., 2015).

Fourth, the hypothesized structural equation model was tested in Amos. Physical exercise volume was specified as an observed exogenous variable, self-compassion was modeled as a second-order latent construct indicated by six first-order dimensions, and body appreciation and psychological flourishing were modeled as latent constructs. The structural model examined the direct association between physical exercise volume and psychological flourishing, as well as the indirect associations via self-compassion, body appreciation, and the serial association involving self-compassion and body appreciation. Model fit was evaluated using the same fit criteria as those applied in the CFA. Bias-corrected bootstrap procedures with 2,000 resamples were used to test the direct, total indirect, specific indirect, and serial indirect associations. Bootstrap confidence intervals were used because indirect associations often do not follow a normal sampling distribution. An indirect association was considered statistically significant when the 95% bias-corrected confidence interval did not include zero (Preacher and Hayes, 2008). The term mediation was used in a statistical sense and did not imply temporal or causal mediation.

Fifth, LPA was conducted in Mplus 8.3 to identify heterogeneous profiles of students' positive self–body relationship. The profile indicators included self-kindness, non-self-judgment, common humanity, non-isolation, mindfulness, non-over-identification, and body appreciation. The original negative self-compassion dimensions were reverse-scored before analysis so that higher scores consistently reflected more adaptive self-compassion-related characteristics. Models with one to eight profiles were estimated using robust maximum likelihood estimation. Multiple random starts were specified to reduce the likelihood of retaining a local maximum solution, and the replication of the best loglikelihood value was checked when evaluating model stability. Model selection was based on a combination of statistical fit, classification quality, profile size, parsimony, and substantive interpretability. Specifically, the Akaike information criterion (AIC), Bayesian information criterion (BIC), sample-size-adjusted Bayesian information criterion (aBIC), entropy, Lo–Mendell–Rubin adjusted likelihood ratio test (LMR), bootstrap likelihood ratio test (BLRT), profile proportions, and average posterior probabilities were examined. Lower AIC, BIC, and aBIC values were interpreted as indicating better relative model fit, whereas higher entropy values indicated better classification accuracy. Significant LMR and BLRT values suggested that the k-profile model provided a better fit than the k – 1 profile model (Lo et al., 2001; Nylund et al., 2007).

The final profile solution was not selected solely according to the lowest information criteria, because information criteria may continue to decrease as additional profiles are extracted. Instead, the retained model was selected by balancing statistical fit, classification quality, average posterior probabilities, adequate profile size, replication of the best loglikelihood, parsimony, and theoretical interpretability. Particular attention was given to the comparison between the four-profile and five-profile solutions. The five-profile solution was inspected to determine whether it produced a substantively distinct additional profile or mainly subdivided an existing profile without clear theoretical meaning. This direct comparison was used to support a transparent and parsimonious profile selection process. For graphical presentation and profile interpretation, the estimated indicator means were transformed into z scores, with positive values indicating scores above the sample mean and negative values indicating scores below the sample mean.

Sixth, after the optimal profile solution was retained, auxiliary-variable analyses were conducted in Mplus. The R3STEP procedure was used to examine whether physical exercise volume was associated with latent profile membership while accounting for classification uncertainty. R3STEP is appropriate for examining associations between covariates and latent profile membership because it preserves the measurement structure of the latent profile model and adjusts for classification uncertainty (Vermunt, 2010; Asparouhov and Muthén, 2014a). Physical exercise volume was treated as the focal covariate in this analysis. The results were reported as multinomial logistic regression coefficients, standard errors, z values, odds ratios, 95% confidence intervals for odds ratios, and *p* values. Odds ratios greater than 1 indicated higher odds of membership in the target profile relative to the reference profile as physical exercise volume increased, whereas odds ratios below 1 indicated lower odds.

Finally, the BCH method was used to compare psychological flourishing across latent profiles while accounting for classification uncertainty. The BCH approach is recommended for examining distal outcomes in mixture models because it allows the latent profile solution to remain unchanged while estimating classification-error-adjusted outcome means (Bolck et al., 2004; Asparouhov and Muthén, 2014b). In the present study, psychological flourishing was specified as the distal outcome. BCH-adjusted means, standard errors, overall Wald tests, and pairwise comparisons were reported. When Mplus did not provide direct confidence intervals for BCH-adjusted distal outcome means, 95% confidence intervals were calculated as BCH mean ± 1.96 × standard error. Pairwise comparison letters were assigned according to the BCH pairwise tests, with different letters indicating statistically significant differences between profiles and the same letter indicating no statistically significant difference at *p* < 0.05.

## 3 Results

### 3.1 Common method bias test

Because all data were collected using self-report questionnaires at a single time point, common method bias was examined before conducting the main analyses. Harman's single-factor test was first performed using an unrotated exploratory factor analysis. All item-level indicators were included in this analysis, including the three original indicators of physical exercise volume. The results showed that nine factors had eigenvalues greater than 1, accounting for 66.83% of the total variance. The first factor explained 32.38% of the variance, which was below the commonly used threshold of 40%. This result suggested that no single factor accounted for the majority of covariance among the observed indicators.

In addition, the confirmatory factor analysis results provided further evidence against serious common method bias. As shown in Table 2, the hypothesized eight-factor model demonstrated excellent fit to the data, χ^2^ = 1,123.755, df = 874, χ^2^/df = 1.286, CFI = 0.996, TLI = 0.996, SRMR = 0.015, RMSEA = 0.011. In contrast, the one-factor model showed poor fit, χ^2^ = 27,558.086, df = 902, χ^2^/df = 30.552, CFI = 0.557, TLI = 0.535, SRMR = 0.107, RMSEA = 0.111. The fit of the eight-factor model was also clearly superior to that of the alternative models in which theoretically distinct constructs were combined.

**Table 2:** Comparative fit indices for competing measurement models.

Model	Factor structure	χ^2^	df	χ^2^/df	CFI	TLI	SRMR	RMSEA
Eight-factor model	SK, NSJ, CH, NIS, MI, NOI, BA, PF	1,123.755	874	1.286	0.996	0.996	0.015	0.011
Seven-factor model	SK + CH, NSJ, NIS, MI, NOI, BA, PF	3,290.866	881	3.735	0.960	0.957	0.035	0.034
Six-factor model A	CSR, NSJ, NIS, NOI, BA, PF	5,311.740	887	5.988	0.926	0.922	0.038	0.046
Six-factor model B	SK, CH, MI, RUSR, BA, PF	5,044.758	887	5.687	0.931	0.926	0.034	0.044
Four-factor model	CSR, RUSR, BA, PF	9,225.349	896	10.296	0.861	0.854	0.049	0.062
Three-factor model	SC, BA, PF	10,708.580	899	11.912	0.837	0.828	0.050	0.067
Two-factor model	SC + BA, PF	19,788.910	901	21.963	0.686	0.670	0.084	0.093
One-factor model	SC + BA + PF	27,558.086	902	30.552	0.557	0.535	0.107	0.111

SK, self-kindness; NSJ, non-self-judgment; CH, common humanity; NIS, non-isolation; MI, mindfulness; NOI, non-over-identification; CSR, compassionate self-responding, including self-kindness, common humanity, and mindfulness; RUSR, reduced uncompassionate self-responding, including non-self-judgment, non-isolation, and non-over-identification; SC, self-compassion; BA, body appreciation; PF, psychological flourishing. The negative self-compassion dimensions were reverse-scored so that higher scores consistently indicated higher self-compassion. PEV was not included in the confirmatory factor analysis because it was computed as an index score based on exercise intensity, duration, and frequency rather than modeled as a reflective latent construct. CFI, comparative fit index; TLI, Tucker–Lewis index; SRMR, standardized root mean square residual; RMSEA, root mean square error of approximation.

To further evaluate potential common method bias, full collinearity VIFs were calculated. At the construct-score level, including physical exercise volume, self-compassion, body appreciation, and psychological flourishing, VIF values ranged from 1.246 to 1.591. As an additional dimension-level check aligned with the latent profile indicators, self-compassion was separated into its six dimensions, and the VIF values ranged from 1.246 to 1.816. All values were below the conservative threshold of 3.3. Taken together, the Harman's single-factor test, competing CFA models, and full collinearity VIF results suggested that common method bias was unlikely to constitute a serious threat to the observed associations.

### 3.2 Preliminary analyses

Descriptive statistics, reliability estimates, convergent validity indices, and bivariate correlations are presented in Table 3. Physical exercise volume showed a mean score of 39.905 (SD = 31.600). Because PARS-3 uses a multiplicative scoring method, the distribution of physical exercise volume was further inspected. Scores ranged from 0 to 100, with a median of 32.00 and an interquartile range of 12.00–64.00. A total of 171 participants scored 0, accounting for 7.12% of the sample. Skewness was 0.473, and excess kurtosis was −1.003. These results suggested that physical exercise volume showed mild positive skewness but no pronounced floor concentration.

**Table 3:** Descriptive statistics, reliability, convergent validity, and correlations among the study variables.

Variable	*M*	SD	Std. loading range	ω	CR	AVE	PEV	SK	NSJ	CH	NIS	MI	NOI	BA	PF
PEV	39.905	31.600	—	—	—	—	—								
SK	3.394	0.989	0.672–0.872	0.919	0.891	0.622	0.279	0.789							
NSJ	3.501	0.922	0.693–0.850	0.906	0.872	0.577	0.290	0.525	0.760						
CH	3.520	0.912	0.671–0.779	0.886	0.829	0.549	0.283	0.517	0.510	0.741					
NIS	3.802	0.923	0.726–0.773	0.895	0.844	0.575	0.286	0.520	0.519	0.530	0.758				
MI	3.626	0.886	0.699–0.783	0.892	0.839	0.567	0.286	0.489	0.493	0.470	0.497	0.753			
NOI	3.514	0.888	0.744–0.766	0.896	0.845	0.577	0.267	0.478	0.496	0.482	0.481	0.459	0.760		
BA	3.330	0.958	0.715–0.799	0.947	0.938	0.602	0.329	0.425	0.430	0.394	0.407	0.394	0.398	0.776	
PF	3.626	0.923	0.733–0.816	0.933	0.918	0.584	0.360	0.324	0.355	0.330	0.337	0.340	0.341	0.391	0.764

PEV, physical exercise volume; SK, self-kindness; NSJ, non-self-judgment; CH, common humanity; NIS, non-isolation; MI, mindfulness; NOI, non-over-identification; BA, body appreciation; PF, psychological flourishing; M, mean; SD, standard deviation; ω, McDonald's omega; CR, composite reliability; AVE, average variance extracted. NSJ, NIS, and NOI were calculated after reverse scoring the original self-judgment, isolation, and over-identification items, respectively, so that higher scores consistently indicate greater self-compassion. Values below the diagonal represent Pearson correlation coefficients. The diagonal values in bold represent the square root of AVE. PEV was treated as an observed index score and was therefore not evaluated for standardized factor loadings, McDonald's ω, CR, or AVE. For the second-order construct of self-compassion, the standardized factor loadings of its six first-order dimensions ranged from 0.732 to 0.791, with McDonald's ω = 0.944, CR = 0.893, and AVE = 0.582. All bivariate correlations were statistically significant at p < 0.01.

The means of the psychological variables ranged from 3.330 to 3.802, suggesting that participants generally reported moderate to relatively high levels of self-compassion, body appreciation, and psychological flourishing. Given that the Flourishing Scale was administered using an adapted five-point response format, its psychometric performance was examined separately. The adapted five-point FS showed excellent internal consistency, McDonald's ω = 0.933. A single-factor CFA of the eight FS items supported the unidimensional structure of the adapted scale, χ^2^/df = 3.478, CFI = 0.995, TLI = 0.994, SRMR = 0.011, RMSEA = 0.032. Standardized factor loadings ranged from 0.733 to 0.816.

The measurement properties of the reflective constructs were satisfactory. Standardized factor loadings ranged from 0.671 to 0.872 across the first-order constructs. McDonald's ω values ranged from 0.886 to 0.947, and composite reliability values ranged from 0.829 to 0.938, exceeding the recommended threshold of 0.70. The AVE values ranged from 0.549 to 0.622, indicating adequate convergent validity. For the second-order construct of self-compassion, the standardized factor loadings of its six first-order dimensions ranged from 0.732 to 0.791, with McDonald's ω = 0.944, CR = 0.893, and AVE = 0.582, further supporting the reliability and convergent validity of the higher-order construct.

Pearson correlation analyses showed that all study variables were significantly and positively correlated with one another (*p* < 0.01). Physical exercise volume was positively correlated with the six dimensions of self-compassion, with correlations ranging from 0.267 to 0.290. Physical exercise volume was also positively associated with body appreciation (*r* = 0.329, *p* < 0.01) and psychological flourishing (*r* = 0.360, *p* < 0.01). Body appreciation was positively correlated with psychological flourishing (*r* = 0.391, *p* < 0.01). This pattern was consistent with the hypothesized positive associations among physical exercise volume, self-compassion, body appreciation, and psychological flourishing.

Discriminant validity was evaluated using both the Fornell–Larcker criterion and the heterotrait–monotrait ratio of correlations. As shown in Table 3, the square root of AVE for each reflective construct was greater than its correlations with other constructs, supporting discriminant validity. In addition, as shown in Table 4, all HTMT values ranged from 0.358 to 0.635, which were below the conservative threshold of 0.85. These results supported adequate discriminant validity among the reflective constructs.

**Table 4:** Discriminant validity based on the heterotrait-monotrait ratio of correlations.

Variable	SK	NSJ	CH	NIS	MI	NOI	BA	PF
SK	—							
NSJ	0.596	—						
CH	0.602	0.600	—					
NIS	0.600	0.607	0.635	—				
MI	0.568	0.577	0.563	0.591	—			
NOI	0.551	0.579	0.577	0.570	0.545	—		
BA	0.466	0.476	0.447	0.458	0.444	0.448	—	
PF	0.358	0.398	0.380	0.384	0.388	0.388	0.421	—

SK, self-kindness; NSJ, non-self-judgment; CH, common humanity; NIS, non-isolation; MI, mindfulness; NOI, non-over-identification; BA, body appreciation; PF, psychological flourishing. Values below the diagonal represent the heterotrait–monotrait ratio of correlations. PEV was not included in the HTMT analysis because it was calculated as an observed index score rather than modeled as a reflective latent construct. HTMT values below 0.85 indicate adequate discriminant validity under the conservative criterion; values below 0.90 may also be considered acceptable under the liberal criterion.

### 3.3 Structural model and statistical indirect associations

**Figure 2:**
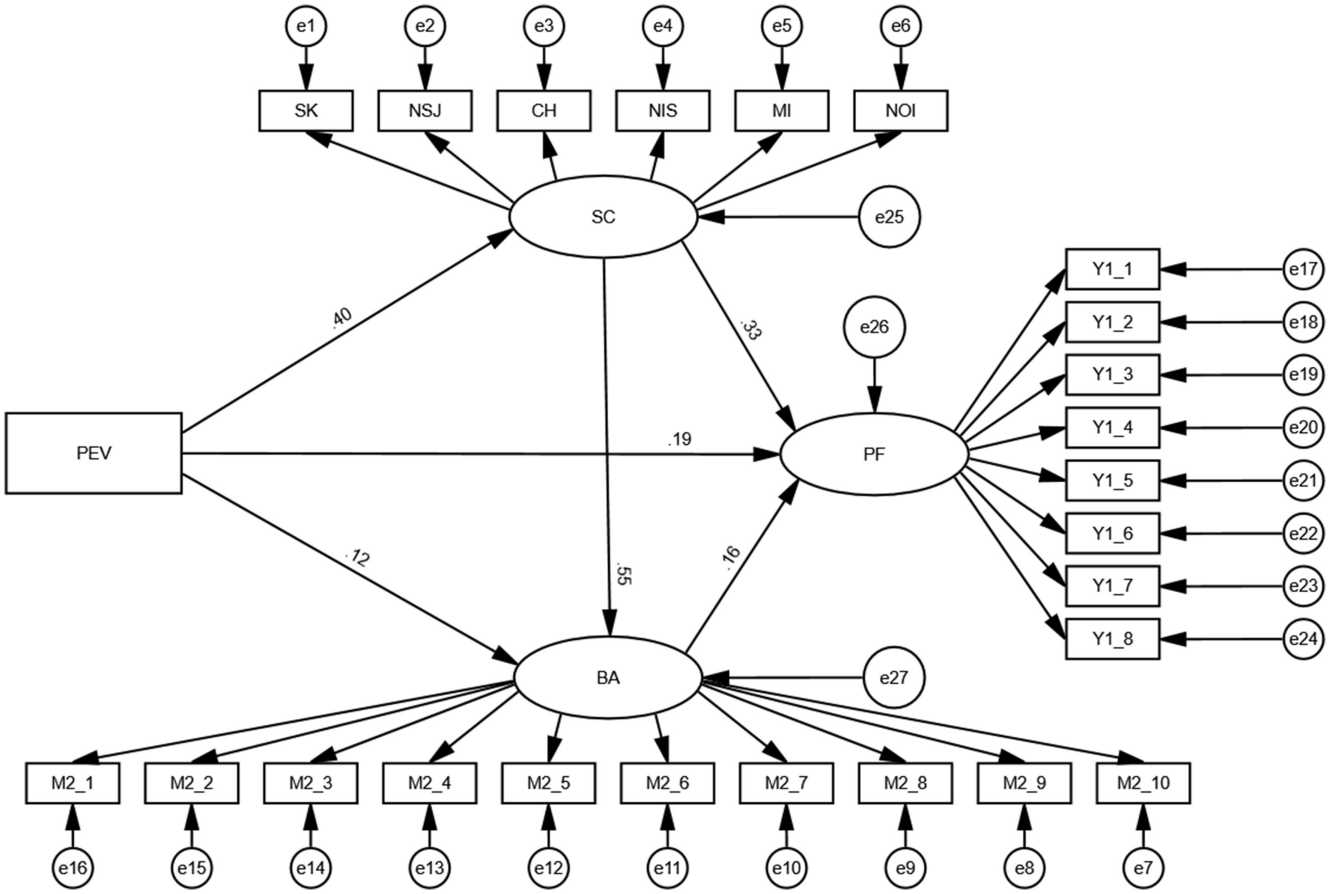
Standardized structural model of direct and serial indirect associations. PEV, physical exercise volume; SC, self-compassion; BA, body appreciation; PF, psychological flourishing. PEV was modeled as an observed exogenous variable. SC was modeled as a second-order latent construct indicated by six first-order dimensions: self-kindness, non-self-judgment, common humanity, non-isolation, mindfulness, and non-over-identification. BA and PF were modeled as latent constructs. Values on paths are standardized coefficients.

The hypothesized structural equation model was then tested to examine the direct and indirect associations among physical exercise volume, self-compassion, body appreciation, and psychological flourishing. The model demonstrated excellent fit to the data: χ^2^/df = 1.491, CFI = 0.996, TLI = 0.996, SRMR = 0.016, and RMSEA = 0.014. These results indicated that the proposed structural model adequately represented the observed data. As shown in Figure 2, all hypothesized structural paths were statistically significant at *p* < 0.05. Physical exercise volume was positively associated with psychological flourishing (β = 0.19), self-compassion (β = 0.40), and body appreciation (β = 0.12). Self-compassion was positively associated with both body appreciation (β = 0.55) and psychological flourishing (β = 0.33). Body appreciation was also positively associated with psychological flourishing (β = 0.16). This pattern was consistent with the proposed model, indicating that students with higher physical exercise volume tended to report higher self-compassion, greater body appreciation, and higher psychological flourishing.

To further examine the statistical indirect associations, bias-corrected bootstrap analyses were conducted with 2,000 resamples. The results are presented in Table 5. The direct association between physical exercise volume and psychological flourishing was significant, β = 0.191, Boot SE = 0.021, 95% CI [0.146, 0.230], accounting for 50.93% of the total association and consistent with H1. The total indirect association was also significant, β = 0.184, Boot SE = 0.012, 95% CI [0.162, 0.210], accounting for 49.07% of the total association. Regarding the specific indirect associations, the indirect association via self-compassion was significant, β = 0.130, Boot SE = 0.013, 95% CI [0.107, 0.159], accounting for 34.67% of the total association and consistent with H2. The indirect association via body appreciation was also significant, β = 0.019, Boot SE = 0.004, 95% CI [0.011, 0.029], accounting for 5.07% of the total association and consistent with H3. The serial indirect association via self-compassion and body appreciation was significant, β = 0.035, Boot SE = 0.006, 95% CI [0.023, 0.048], accounting for 9.33% of the total association and consistent with H4. Among the indirect associations, the association via self-compassion was the largest, whereas the body appreciation-specific association was smaller but remained statistically significant. Overall, these results indicated that physical exercise volume was associated with psychological flourishing both directly and indirectly through self-compassion, body appreciation, and their serial association.

**Table 5:** Bias-corrected bootstrap results for direct, specific indirect, and serial indirect associations in the structural model.

Hypothesis	Path	β	Boot SE	Boot LLCI	Boot ULCI	Ratio (%)
H1	Direct association: PEV → PF	0.191	0.021	0.146	0.230	50.93
H2–H4	Total indirect association	0.184	0.012	0.162	0.210	49.07
H2	PEV → SC → PF	0.130	0.013	0.107	0.159	34.67
H3	PEV → BA → PF	0.019	0.004	0.011	0.029	5.07
H4	PEV → SC → BA → PF	0.035	0.006	0.023	0.048	9.33
—	Total association: PEV → PF	0.375	0.018	0.337	0.410	100.00

PEV, physical exercise volume; SC, self-compassion; BA, body appreciation; PF, psychological flourishing. Boot SE, bootstrap standard error; Boot LLCI, lower limit of the 95% bias-corrected confidence interval; Boot ULCI, upper limit of the 95% bias-corrected confidence interval. Ratio (%) represents the proportion of each direct or indirect association relative to the total association. Confidence intervals that do not include zero indicate statistically significant associations. Bootstrap estimates were based on 2,000 resamples.

### 3.4 Latent profile analysis

Latent profile analysis was conducted using seven indicators: self-kindness, non-self-judgment, common humanity, non-isolation, mindfulness, non-over-identification, and body appreciation. To facilitate profile interpretation and graphical presentation, the estimated indicator means were standardized into z scores, such that values above zero indicate scores higher than the sample mean and values below zero indicate scores lower than the sample mean.

One- to eight-profile solutions were estimated and compared using multiple criteria, including information criteria, entropy, likelihood ratio tests, profile size, classification precision, model stability, parsimony, and theoretical interpretability. As shown in Table 6, AIC, BIC, and aBIC values decreased as the number of profiles increased, and both the LMR and BLRT tests were significant across the tested solutions. However, because information criteria often continue to decrease as additional profiles are extracted, the final model was not selected solely on the basis of the lowest information criteria. Instead, the retained solution was selected by balancing statistical fit with classification quality and substantive meaning.

**Table 6:** Model fit indices for one- to eight-profile latent profile solutions.

No. of profiles	AIC	BIC	aBIC	Entropy	LMR (*p*)	BLRT (*p*)	Profile proportions
1	45,090.992	45,171.963	45,127.482	—	—	—	1.000
2	39,557.481	39,684.721	39,614.823	0.869	< 0.001	< 0.001	0.44/0.56
3	38,748.393	38,921.902	38,826.585	0.787	< 0.001	< 0.001	0.28/0.38/0.34
4	38,548.956	38,768.734	38,648.000	0.799	< 0.001	< 0.001	0.27/0.38/0.21/0.14
5	38,391.545	38,657.593	38,511.441	0.759	0.002	< 0.001	0.132/0.21/0.20/0.15/0.31
6	38,233.080	38,545.397	38,373.827	0.776	< 0.001	< 0.001	0.16/0.16/0.11/0.10/0.20/0.27
7	38,131.478	38,490.064	38,293.076	0.783	0.031	< 0.001	0.16/0.15/0.10/0.12/0.28/0.08/0.11
8	37,964.669	38,369.523	38,147.118	0.799	< 0.001	< 0.001	0.15/0.09/0.07/0.08/0.12/0.10/0.12/0.28

AIC, Akaike information criterion; BIC, Bayesian information criterion; aBIC, sample-size-adjusted Bayesian information criterion; LMR, Lo–Mendell–Rubin adjusted likelihood ratio test; BLRT, bootstrap likelihood ratio test. Lower AIC, BIC, and aBIC values indicate better relative model fit, whereas higher entropy values indicate greater classification accuracy. Significant LMR and BLRT values indicate that the k-profile model fits significantly better than the k – 1 profile model. Profile proportions are based on estimated latent profile probabilities. Entropy, LMR, and BLRT are not applicable to the one-profile model. The retained profile solution was selected based on statistical fit indices, classification quality, profile size, average posterior probabilities, stability, parsimony, and substantive interpretability.

The four-profile solution was retained as the final model for several reasons. First, the four-profile solution showed acceptable classification quality, with entropy = 0.799. Although the five-profile solution showed lower information criteria, its entropy decreased to 0.759, indicating weaker classification clarity than the four-profile solution. Second, the four-profile solution produced adequate profile sizes, with the smallest profile representing 14.16% of the sample, and the average posterior probabilities for the retained profiles ranged from 0.800 to 0.937, indicating acceptable-to-good classification precision. Third, inspection of the five-profile solution indicated that the additional profile mainly subdivided an existing moderate group by level rather than representing a substantively distinct self–body configuration. Fourth, the four-profile solution yielded a clear and parsimonious configuration of positive self–body relationship, including two globally distinct profiles and two theoretically informative moderate profiles. Finally, the retained four-profile solution was rerun with increased random starts, and the same best loglikelihood and substantively identical profile pattern were obtained, supporting the stability of the retained solution. Therefore, the four-profile solution was selected as the final model.

For the retained four-profile solution, the most likely class membership counts were 645 participants in Profile 1, 921 in Profile 2, 495 in Profile 3, and 340 in Profile 4, corresponding to 26.86%, 38.36%, 20.62%, and 14.16% of the sample, respectively. The average posterior probabilities for the most likely class membership were 0.919 for Profile 1, 0.937 for Profile 2, 0.822 for Profile 3, and 0.800 for Profile 4, indicating acceptable-to-good classification precision.

**Figure 3:**
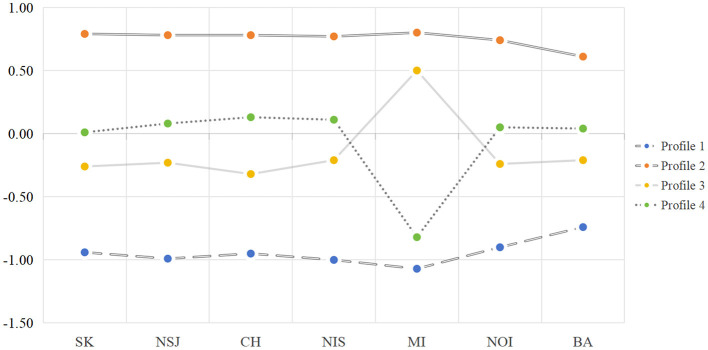
Z-standardized estimated indicator means across the four retained latent profiles. SK, self-kindness; NSJ, non-self-judgment; CH, common humanity; NIS, non-isolation; MI, mindfulness; NOI, non-over-identification; BA, body appreciation. Values represent z-standardized estimated means of the seven profile indicators in the retained four-profile solution. A value of 0 represents the sample mean, positive values indicate scores above the sample mean, and negative values indicate scores below the sample mean. NSJ, NIS, and NOI were reverse-scored so that higher values indicate lower uncompassionate self-responding. Profile 1, low self-compassion–low body appreciation profile; Profile 2, high self-compassion–high body appreciation profile; Profile 3, mindfulness-dominant moderate profile; Profile 4, low-mindfulness moderate profile.

Figure 3 presents the z-standardized estimated means of the seven indicators across the four latent profiles. Profile 1 showed consistently low scores across all indicators, including self-kindness, non-self-judgment, common humanity, non-isolation, mindfulness, non-over-identification, and body appreciation. This profile was therefore labeled the low self-compassion–low body appreciation profile. Profile 2 showed consistently high scores across all indicators and was labeled the high self-compassion–high body appreciation profile. Profile 3 was characterized by generally moderate-to-slightly-low scores on most indicators but a relatively high score on mindfulness; therefore, it was labeled the mindfulness-dominant moderate profile. Profile 4 showed generally moderate scores on most indicators but a notably low score on mindfulness, and was labeled the low-mindfulness moderate profile.

The R3STEP multinomial logistic regression was then conducted to examine whether physical exercise volume was associated with latent profile membership while accounting for classification uncertainty. As shown in Table 7, Profile 4 was used as the reference category. Higher physical exercise volume was associated with significantly lower odds of belonging to Profile 1 rather than Profile 4, B = −0.018, SE = 0.003, *z* = −5.303, *p* < 0.001, OR = 0.982, 95% CI [0.976, 0.989]. Higher physical exercise volume was also associated with significantly higher odds of belonging to Profile 2 rather than Profile 4, B = 0.015, SE = 0.003, *z* = 5.810, *p* < 0.001, OR = 1.015, 95% CI [1.010, 1.020]. For interpretability, these odds ratios correspond approximately to 17% lower odds of belonging to Profile 1 rather than Profile 4 and 16% higher odds of belonging to Profile 2 rather than Profile 4 for every 10-point increase in physical exercise volume. However, physical exercise volume was not significantly associated with membership in Profile 3 relative to Profile 4, B = −0.004, SE = 0.003, *z* = −1.270, *p* = 0.204, OR = 0.996, 95% CI [0.989, 1.002]. These results indicated that greater physical exercise volume was associated with a higher likelihood of belonging to the most adaptive profile and a lower likelihood of belonging to the least adaptive profile, relative to the low-mindfulness moderate profile.

**Table 7:** R3STEP multinomial logistic regression examining the association between physical exercise volume and latent profile membership.

Target profile	Profile characteristics	*n* (%)	B	SE	*z*	OR	95% CI for OR	*p*
Profile 1: low self-compassion–low body appreciation profile	Generally low levels across self-compassion dimensions and body appreciation	645 (26.86%)	−0.018	0.003	−5.303	0.982	[0.976, 0.989]	< 0.001
Profile 2: high self-compassion–high body appreciation profile	High levels across self-compassion dimensions and body appreciation	921 (38.36%)	0.015	0.003	5.810	1.015	[1.010, 1.020]	< 0.001
Profile 3: mindfulness-dominant moderate profile	Moderate self-compassion and body appreciation, with relatively high mindfulness	495 (20.62%)	−0.004	0.003	−1.270	0.996	[0.989, 1.002]	0.204
Reference: profile 4: low-mindfulness moderate profile	Moderate self-compassion and body appreciation, with relatively low mindfulness	340 (14.16%)	—	—	—	—	—	—

PEV, physical exercise volume; OR, odds ratio; CI, confidence interval. Profile 4, the low-mindfulness moderate profile, was used as the reference category. The R3STEP procedure was used to examine the association between PEV and latent profile membership while accounting for classification uncertainty. OR values greater than 1 indicate that higher PEV is associated with higher odds of belonging to the target profile rather than the reference profile, whereas OR values below 1 indicate lower odds of belonging to the target profile rather than the reference profile.

Finally, BCH analyses were performed to compare psychological flourishing across the four latent profiles while accounting for classification uncertainty. As shown in Table 8, the overall Wald test was significant, χ^2^_(3)_ = 522.902, *p* < 0.001, indicating that psychological flourishing differed significantly across profiles. Profile 2 had the highest BCH-adjusted mean of psychological flourishing, M = 4.071, SE = 0.028, 95% CI [4.016, 4.126], whereas Profile 1 had the lowest mean, M = 3.032, SE = 0.036, 95% CI [2.961, 3.103]. Profiles 3 and 4 showed intermediate levels of psychological flourishing, with BCH-adjusted means of 3.527 and 3.684, respectively. Pairwise comparisons indicated that Profile 2 differed significantly from all other profiles and that Profile 1 also differed significantly from all other profiles. Profiles 3 and 4 did not differ significantly from each other, χ^2^ = 3.363, *p* = 0.067. Overall, students in the high self-compassion–high body appreciation profile reported the highest level of psychological flourishing, whereas those in the low self-compassion–low body appreciation profile reported the lowest level. The two moderate profiles showed statistically comparable levels of psychological flourishing.

**Table 8:** BCH comparisons of psychological flourishing across latent profiles.

Profile	BCH mean of PF	SE	95% CI	Pairwise comparison
Profile 1: low self-compassion–low body appreciation profile	3.032	0.036	[2.961, 3.103]	c
Profile 2: high self-compassion–high body appreciation profile	4.071	0.028	[4.016, 4.126]	a
Profile 3: mindfulness-dominant moderate profile	3.527	0.050	[3.429, 3.625]	b
Profile 4: low-mindfulness moderate profile	3.684	0.061	[3.564, 3.804]	b

PF, psychological flourishing; BCH, Bolck–Croon–Hagenaars method; CI, confidence interval. BCH means represent classification-error-adjusted means of psychological flourishing across latent profiles. Pairwise comparison letters were assigned according to the BCH pairwise tests of PF means. Different letters in the Pairwise comparison column indicate statistically significant differences between profiles at p < 0.05, whereas the same letter indicates no statistically significant difference. Specifically, letter a indicates the highest PF group, letter b indicates the intermediate PF group, and letter c indicates the lowest PF group.

## 4 Discussion

### 4.1 Overview of the main findings

The present study examined the association between physical exercise volume and psychological flourishing among Chinese college students by integrating variable-centered and person-centered approaches. The variable-centered results showed that physical exercise volume was positively associated with psychological flourishing, and that this association included significant indirect associations via self-compassion, body appreciation, and their serial association. The person-centered results further showed that students' positive self–body resources were not configured uniformly. Instead, four distinct profiles emerged: a low self-compassion–low body appreciation profile, a high self-compassion–high body appreciation profile, a mindfulness-dominant moderate profile, and a low-mindfulness moderate profile.

Taken together, these findings suggest that the exercise–flourishing linkage among college students should be understood not only as a matter of exercise volume, but also as a matter of how students relate to themselves and their bodies. The structural model clarified average-level indirect associations among physical exercise volume, self-compassion, body appreciation, and flourishing, whereas the latent profile results revealed meaningful heterogeneity in students' self–body configurations. This combined perspective provides a more nuanced understanding of exercise-related positive mental health than either variable-centered or person-centered analysis alone.

### 4.2 Physical exercise volume and psychological flourishing: beyond symptom reduction

Consistent with the positive mental health perspective, physical exercise volume was positively associated with psychological flourishing (Zhang et al., 2026). This finding is meaningful because flourishing reflects more than the absence of psychological distress. It involves perceived meaning, competence, optimism, self-respect, positive relationships, and contribution to others. In this sense, the present result supports the view that physical exercise among college students should not be discussed only as a behavioral strategy for reducing negative symptoms, but also as a behavioral context related to optimal psychological functioning.

For Chinese college students, physical exercise may be embedded in multiple developmental contexts, including academic stress regulation, peer interaction, self-discipline, bodily competence, and identity exploration (Li and Huang, 2024). Therefore, the positive association between physical exercise volume and psychological flourishing may reflect more than physiological activation or energy expenditure. Students with greater exercise volume may also have more opportunities to experience mastery, routine, vitality, bodily agency, and social participation (Wong et al., 2026). These experiences are closely aligned with the functional components of flourishing and may help explain why exercise volume was positively linked to students' positive functioning.

At the same time, the present findings should be interpreted as associations rather than evidence that exercise volume directly produces flourishing. The cross-sectional design does not allow temporal or causal inference. It is also plausible that students with higher flourishing are more likely to participate in physical exercise, maintain exercise routines, or perceive exercise experiences more positively. Therefore, the present result is best understood as evidence of a meaningful positive linkage between exercise volume and flourishing, rather than as evidence of a one-directional causal effect.

### 4.3 Self-compassion as a central self-regulatory resource

Among the indirect associations, self-compassion showed the largest specific indirect association between physical exercise volume and psychological flourishing. This finding is theoretically important because it positions self-compassion as a central self-regulatory and self-relational resource in the exercise–flourishing linkage.

Self-compassion refers to treating oneself with kindness, recognizing difficulties as part of common humanity, and maintaining balanced awareness in the face of stress or inadequacy (Neff, 2003). In the context of physical exercise, students may encounter bodily limitations, fatigue, performance comparison, irregular progress, and self-evaluation. These experiences do not only require behavioral regulation, such as sustaining participation or adjusting effort; they also require self-evaluative regulation, such as interpreting imperfection without harsh self-criticism. The present findings suggest that students with greater exercise volume tended to report higher self-compassion, and students with higher self-compassion tended to report higher psychological flourishing. This pattern is consistent with the idea that flourishing depends not only on external achievements or positive emotions, but also on how individuals respond to imperfection, effort, and difficulty.

The relatively larger indirect association via self-compassion suggests that the exercise–flourishing linkage may be more strongly connected with students' broad self-regulatory and self-relational orientation than with body-specific attitudes alone. Much prior work has focused on self-efficacy, resilience, or meaning as mediating resources in exercise-related wellbeing research (August et al., 2023). The present study adds to this literature by showing that a kind, nonjudgmental, and mindful orientation toward the self is closely tied to the association between exercise volume and flourishing. This may be especially relevant in university settings where students often face academic competition, peer comparison, and self-evaluative pressure. Under such conditions, self-compassion may help students remain engaged in exercise and broader college life without excessive self-criticism.

However, the finding should not be interpreted as showing that exercise necessarily increases self-compassion. Rather, it indicates that physical exercise volume, self-compassion, and flourishing were positively connected in the observed sample. Future longitudinal work is needed to clarify whether changes in exercise participation are followed by changes in self-compassion, whether self-compassion supports sustained exercise behavior, or whether the relationship is reciprocal.

### 4.4 Body appreciation as an embodied psychological resource

Body appreciation also showed a significant indirect association between physical exercise volume and psychological flourishing, although the size of this specific indirect association was smaller than that of self-compassion. This finding supports the theoretical relevance of positive body image in understanding college students' flourishing, while also clarifying its more domain-specific role.

Unlike body dissatisfaction, which focuses on negative evaluation of appearance, body appreciation emphasizes acceptance, respect, gratitude, and care toward the body (Tylka and Wood-Barcalow, 2015b). The present study suggests that students with higher physical exercise volume tended to report greater body appreciation, and students with greater body appreciation tended to report higher psychological flourishing. This pattern is consistent with the view that the body can be a source of competence, agency, and positive self-experience rather than merely an object of appearance evaluation (Piran, 2019; Zhu and Wen, 2025). In this sense, body appreciation may be understood as an embodied psychological resource: it reflects a positive relationship with the body as the basis of action, vitality, participation, and engagement with life.

The smaller size of the physical exercise volume → body appreciation → psychological flourishing association should be interpreted carefully. It does not mean that body appreciation is unimportant. Instead, it may indicate that body appreciation functions as a more body-specific resource, whereas self-compassion reflects a broader orientation toward the self (Cha et al., 2023; Neff, 2023). In the present model, self-compassion was strongly associated with body appreciation, and the serial indirect association through self-compassion and body appreciation accounted for a larger proportion of the total association than the body appreciation-specific association alone. This suggests that body appreciation may be especially meaningful when situated within a broader compassionate self-relation.

The significant serial indirect association therefore provides an important theoretical refinement. Physical exercise volume was associated with psychological flourishing partly through a broader self-compassionate orientation and then through a more specific positive body orientation (Zhu and Wen, 2025). This supports the logic that students' relationship with their bodies may be embedded within their broader relationship with themselves (Geller et al., 2021). In practical terms, this means that exercise programs focused on positive body image may be more useful when they also cultivate self-kindness, reduced self-judgment, and nonappearance-based body value, rather than focusing on body appreciation in isolation.

### 4.5 Heterogeneous profiles of positive self–body relationship

The latent profile analysis extended the variable-centered findings by showing that self-compassion and body appreciation were not distributed in a single uniform pattern across students. Instead, four distinct configurations emerged. This person-centered result is important because it demonstrates that students may have different combinations of self-related and body-related resources, even when the same variables show positive associations at the average level.

The first profile, labeled the low self-compassion–low body appreciation profile, showed consistently low standardized scores across all seven indicators. This profile represented more than one isolated deficit. Rather, it reflected a broad depletion of positive self–body resources, including lower self-kindness, common humanity, mindfulness, non-self-judgment, non-isolation, non-over-identification, and body appreciation. Students in this profile may be especially vulnerable because less adaptive self-relating and less appreciative body-relating appear together. This configuration may make exercise and body-related experiences more likely to be interpreted through self-criticism, comparison, or inadequacy.

The second profile, labeled the high self-compassion–high body appreciation profile, showed the opposite pattern, with consistently high scores across all indicators. This profile can be understood as an integrated positive self–body configuration. Students in this group reported both a compassionate way of relating to themselves and an appreciative way of relating to their bodies. The profile-level pattern is consistent with the variable-centered model, in which self-compassion and body appreciation were positively connected. It also suggests that the most adaptive self–body configuration may involve the co-occurrence of broad self-kindness, reduced self-criticism, mindful awareness, and positive body regard.

The two moderate profiles are especially theoretically informative. Profile 3, the mindfulness-dominant moderate profile, showed a relatively high score on mindfulness but generally moderate-to-slightly-low scores on other indicators. This profile may reflect awareness without sufficient warmth. That is, students in this profile may be relatively able to notice bodily or emotional experiences, but this awareness may not be fully accompanied by self-kindness, common humanity, reduced self-judgment, or body appreciation. This distinction is important because mindfulness is often assumed to be adaptive, but awareness alone may not be sufficient for flourishing when it is not integrated with acceptance, warmth, and a positive body orientation.

Profile 4, the low-mindfulness moderate profile, showed a notably low score on mindfulness but relatively moderate scores on most other indicators. This profile should not be interpreted simply as a global risk group. Although these students reported lower mindfulness, their other self-compassion dimensions and body appreciation were closer to moderate levels. Therefore, low mindfulness alone may not indicate broadly poor self–body functioning. Instead, this profile suggests that students may maintain some degree of self-kindness, reduced self-judgment, common humanity, and body appreciation even when mindful awareness is relatively low.

These two moderate profiles contribute to ongoing discussion about whether self-compassion should be understood mainly as a global construct or as a multidimensional configuration. At the variable-centered level, self-compassion functioned as a coherent higher-order construct in the structural model. At the person-centered level, however, different dimensions of self-compassion combined with body appreciation in distinguishable ways. Thus, the present study shows that global self-compassion is meaningful, but dimensional patterns also carry important psychological information.

### 4.6 Physical exercise volume, profile membership, and psychological flourishing

The R3STEP results provide further insight into how physical exercise volume was associated with different positive self–body profiles. Compared with the low-mindfulness moderate profile, higher physical exercise volume was associated with lower odds of belonging to the low self-compassion–low body appreciation profile and higher odds of belonging to the high self-compassion–high body appreciation profile. This pattern suggests that exercise volume was most clearly linked to the contrast between broadly adaptive and broadly less adaptive self–body configurations (Zhu and Wen, 2025).

Although the odds ratios per one-point increase in physical exercise volume were small, their practical meaning becomes clearer when interpreted across a larger and more meaningful score difference. For the low self-compassion–low body appreciation profile, the odds ratio of 0.982 corresponds to approximately 0.83 for every 10-point increase in physical exercise volume, indicating about 17% lower odds of belonging to this profile relative to the low-mindfulness moderate profile. For the high self-compassion–high body appreciation profile, the odds ratio of 1.015 corresponds to approximately 1.16 for every 10-point increase, indicating about 16% higher odds of belonging to this profile relative to the low-mindfulness moderate profile. These estimates suggest that the association between exercise volume and profile membership, although modest at the unit-score level, has meaningful applied relevance when interpreted across the broader range of exercise volume.

However, physical exercise volume was not significantly associated with membership in the mindfulness-dominant moderate profile relative to the low-mindfulness moderate profile. This non-significant result is theoretically meaningful. It suggests that exercise volume alone may not explain the more subtle distinction between different moderate configurations of self-compassion and body appreciation. The two moderate profiles differed mainly in the relative position of mindfulness, whereas their overall levels of several other indicators were closer to the sample mean. Therefore, whether students show a mindfulness-dominant or low-mindfulness moderate profile may depend on factors not captured by exercise volume, such as exercise type, motivational climate, reflective attention during exercise, stress level, personality, or prior experience with mindfulness-related practices (Lecuona et al., 2024).

The BCH results further strengthen the person-centered interpretation. Psychological flourishing was highest in the high self-compassion–high body appreciation profile and lowest in the low self-compassion–low body appreciation profile. This indicates that flourishing was most favorable among students whose self-related and body-related resources were consistently high, and least favorable among students whose resources were consistently low (Wu et al., 2020). Importantly, Profiles 3 and 4 did not differ significantly in psychological flourishing. Although Profile 4 showed lower mindfulness, it had relatively moderate scores on other indicators, whereas Profile 3 showed relatively high mindfulness but lower scores on several other indicators. This pattern suggests that psychological flourishing may depend more on the integrated configuration of positive self–body resources than on mindfulness alone (Marcovski, 2024).

This finding helps avoid an overly simplified interpretation of mindfulness. Mindfulness is important, but a relatively high mindfulness score did not correspond to significantly higher flourishing when other self-compassion and body appreciation indicators were not similarly elevated. Conversely, lower mindfulness did not necessarily correspond to lower flourishing when other indicators remained moderate. Thus, the profile results point to the importance of balance and integration among self-kindness, reduced self-judgment, common humanity, body appreciation, and mindful awareness (Wu et al., 2020; Zhu and Wen, 2025).

### 4.7 Theoretical implications

The present study offers several theoretical implications. First, it extends research on physical exercise and college student mental health by placing flourishing, rather than distress reduction, at the center of analysis. This shift is not merely a change in outcome variable. It reflects a broader theoretical move from a deficit-oriented model to a positive functioning model. The findings suggest that physical exercise volume is meaningfully associated with students' perceived success in important life domains, and that this association is intertwined with how students relate to themselves and their bodies.

Second, the findings contribute to self-compassion theory by showing that self-compassion is not only associated with psychological wellbeing in general, but also plays a prominent role in an embodied behavioral context. Physical exercise involves effort, bodily feedback, comparison, discipline, and sometimes frustration. The present results suggest that self-compassion is a theoretically relevant construct for understanding how students' exercise-related experiences are linked to flourishing. In this respect, self-compassion should not be viewed only as an emotion-regulation resource in stressful situations, but also as a self-relational and self-regulatory resource connected to positive development.

Third, the findings add to positive body image theory by positioning body appreciation as part of a broader positive self–body system. Body appreciation showed its own indirect association with psychological flourishing, but it was also closely connected with self-compassion. This supports the view that positive body image is not simply the absence of dissatisfaction or appearance concern. Rather, it is a positive psychological resource that can be integrated with broader self-acceptance, self-kindness, and psychological functioning. The relatively smaller body appreciation-specific association also helps clarify the theoretical boundary of body appreciation: it appears to be important, but its meaning may be strengthened when embedded within a broader compassionate self-orientation.

Fourth, the study contributes to the dialogue between variable-centered and person-centered approaches. The structural model showed how physical exercise volume, self-compassion, body appreciation, and flourishing were associated on average. The LPA results showed that these resources were configured differently across students. This dual approach is theoretically valuable because it prevents two forms of oversimplification: assuming that all students follow the same average pattern, or assuming that profile differences can be understood without considering structural associations among constructs. By integrating both approaches, the study provides a more nuanced account of the exercise–self–body–flourishing linkage.

Finally, the present findings have implications for understanding positive self–body resources in the Chinese college context. Chinese college students may experience academic competition, peer evaluation, collective expectations, and body-related social pressures. In this context, self-compassion and body appreciation may be particularly relevant resources for positive functioning because they help students relate to effort, imperfection, and bodily experience with less harshness and more respect. The distinction between the mindfulness-dominant moderate profile and the low-mindfulness moderate profile also raises a theoretical question about the internal structure of self-compassion. Although self-compassion can be modeled as a higher-order construct, its components may have different meanings when examined at the person level. Future theory should therefore pay more attention to configurations among self-compassion dimensions, rather than treating all components as uniformly high or low within individuals.

### 4.8 Practical implications

The present findings also have implications for university physical education, student affairs, and mental health services. First, campus exercise programs should not focus only on increasing exercise volume. Although physical exercise volume was positively associated with flourishing and with more adaptive profile membership, the results also suggest that psychological resources related to self-compassion and body appreciation are important (Cox et al., 2019). Therefore, exercise programs may be more beneficial for positive student development when they are embedded in a supportive psychological climate. In practice, this means that universities should promote exercise participation while also reducing overly evaluative, appearance-focused, ranking-based, or shame-based exercise environments.

For students resembling the low self-compassion–low body appreciation profile, interventions should avoid highly evaluative, appearance-focused, or ranking-based exercise climates (August et al., 2023). These students may be particularly sensitive to self-criticism and negative body evaluation. Programs for this group could emphasize gradual participation, mastery experiences, bodily functionality, self-kindness, and nonjudgmental reflection. For example, instructors could encourage students to notice what their bodies can do, set personally meaningful goals, and respond to setbacks with patience rather than self-blame. Such practices may help students experience exercise as a context of self-care rather than self-evaluation.

For students resembling the high self-compassion–high body appreciation profile, the practical goal may be maintenance and enrichment rather than remediation. These students already show a relatively adaptive self–body configuration and the highest flourishing level. Universities could provide diverse exercise opportunities, peer-support roles, and autonomy-supportive activities that allow these students to maintain engagement without turning exercise into perfectionistic self-monitoring or appearance management (Seekis et al., 2020). These students may also serve as positive peer models in inclusive and supportive exercise environments.

For students in the mindfulness-dominant moderate profile, the findings suggest that mindful awareness alone may not be sufficient if other self-compassion and body appreciation indicators remain relatively low. Programs for this group could focus on translating awareness into self-kindness, reduced self-judgment, and appreciation of the body. Mindful movement, reflective journaling after exercise, and body functionality exercises may be useful components, especially when they explicitly connect attention to the body with acceptance and respect (Granfield et al., 2023; Stern and Engeln, 2018). The practical emphasis should be on helping students move from noticing experience to responding to experience with warmth and appreciation.

For students in the low-mindfulness moderate profile, interventions could focus on helping students notice bodily cues, emotional states, and recovery needs during exercise (Rudaz et al., 2023). However, because this profile did not differ significantly from the mindfulness-dominant moderate profile in psychological flourishing, low mindfulness should not be treated as a standalone risk marker. Instead, support should consider the whole configuration of self-compassion and body appreciation. For this group, brief body-awareness activities, recovery education, and post-exercise reflection may be useful, provided that they are implemented in a non-stigmatizing and non-labeling way.

These findings also suggest that universities could use brief assessments of self-compassion and body appreciation to inform more targeted health promotion. The purpose should not be to label students rigidly, but to identify different support needs. A profile-informed approach may help universities move from one-size-fits-all exercise promotion toward more differentiated programs that connect physical activity with positive self-development. In this sense, university-based exercise promotion should aim not only to increase how much students move, but also to improve the psychological climate in which movement is experienced.

### 4.9 Limitations and future directions

Several limitations should be considered. First, the study used a cross-sectional design. Although the hypothesized serial indirect association model was theoretically grounded, the temporal ordering among physical exercise volume, self-compassion, body appreciation, and psychological flourishing cannot be established from the present data. The observed indirect associations should therefore be interpreted as statistical associations rather than causal mechanisms. Future studies should use longitudinal, cross-lagged, experience-sampling, or intervention designs to examine whether changes in exercise volume are followed by changes in self-compassion, body appreciation, and flourishing, whether self-compassion and body appreciation support sustained exercise behavior, or whether these associations are reciprocal.

Second, although procedural and statistical remedies were used to evaluate common method bias, all focal variables were collected through self-report measures at a single time point. Harman's single-factor test, competing CFA models, and full collinearity VIFs suggested that common method bias was unlikely to constitute a serious threat to the observed associations. Nevertheless, common method variance cannot be fully ruled out. Future research should consider multi-source, multi-method, or temporally separated designs, such as combining self-report measures with objective physical activity monitoring, peer or instructor ratings, diary methods, or repeated assessments over time.

Third, physical exercise volume was measured using the PARS-3 index based on intensity, duration, and frequency. This measure is appropriate for assessing overall exercise volume in Chinese samples, but it does not distinguish among exercise types, contexts, motivations, or subjective exercise experiences. In addition, because PARS-3 uses a multiplicative scoring method, very low or zero values on one component can influence the total score. The present distributional inspection indicated only a modest proportion of zero scores, but the scoring structure should still be considered when interpreting exercise volume. The present R3STEP results also showed that physical exercise volume differentiated the high and low self–body profiles but did not distinguish the two moderate profiles. This suggests that exercise quality may matter in addition to exercise quantity. Future research should examine whether autonomy-supportive exercise environments, team-based activities, mindful movement, competitive sports, aesthetic exercise, or appearance-focused fitness contexts are differently associated with self-compassion, body appreciation, and profile membership.

Fourth, the Flourishing Scale was administered using a five-point response format rather than its original seven-point format. Although the adapted five-point FS showed excellent internal consistency and satisfactory factorial validity in the present sample, the response-format adaptation should be acknowledged. Changing the response format may influence response variability, scale sensitivity, mean-level comparability, and measurement invariance across studies. Future studies could directly compare five-point and seven-point versions of the FS in Chinese college student samples and examine whether response format affects mean levels, factor structure, measurement invariance, or associations with exercise and self–body variables.

Fifth, demographic variables were reported descriptively but were not included as covariates in the focal models. This decision was consistent with the theory-driven focus of the study, which centered on physical exercise volume, self-compassion, body appreciation, psychological flourishing, and self–body profiles. Nevertheless, demographic characteristics such as gender, age, grade, academic discipline, residence background, and university type may shape exercise experiences, body-related social pressures, and psychological flourishing. Future studies should examine whether the observed associations and latent profiles differ across demographic groups, or whether demographic variables moderate the relationships among exercise volume, self-compassion, body appreciation, and flourishing.

Sixth, although the four-profile solution was selected based on classification quality, profile size, stability, parsimony, and substantive interpretability, the information criteria continued to decrease as the number of profiles increased. This is common in mixture modeling, but it means that the retained solution should be understood as the most interpretable and parsimonious representation of the present data rather than the only possible classification. Future research should replicate the four-profile solution in independent samples, test its stability across gender, grade, university type, and region, and examine whether similar profiles emerge in other cultural contexts.

Seventh, the sample was drawn from universities in Sichuan Province. Although the sample included students from different types of universities, disciplines, grades, and residence backgrounds, it may not fully represent all Chinese college students. Regional differences in campus sport culture, academic pressure, body norms, and access to exercise facilities may shape the associations observed in this study. Future studies should use multi-province or nationally representative samples to examine the generalizability of the findings.

Eighth, the negative self-compassion dimensions were reverse-scored so that higher scores represented lower uncompassionate self-responding. This scoring approach allowed all indicators to be interpreted in the same adaptive direction and made the profile figure clearer. However, reverse-scored dimensions may still carry distinct psychological meanings. For example, low over-identification is not identical to high mindfulness, and low isolation is not identical to high common humanity. Future studies could compare alternative self-compassion measurement models, such as six-factor, two-factor, and bifactor models, and examine whether profile structures differ when positive and negative self-compassion components are modeled separately.

Finally, the present study focused on self-compassion and body appreciation, but other variables may help explain why exercise volume was associated with flourishing and profile membership. These may include exercise motivation, perceived physical competence, social support, body mass index, body functionality, appearance pressure, social physique anxiety, sleep quality, and academic stress. Future studies could also consider broader educational psychological resources, such as emotional intelligence, which has been identified as an important and growing topic in educational research (Solih et al., 2024). In addition, because students' self–body experiences are increasingly shaped by online and social media environments, future research may examine how digital social contexts are related to self-compassion, body appreciation, physical activity, and flourishing; related work has highlighted the relevance of social media environments for mental health in other populations (Tan et al., 2024). Including these variables and contexts in future studies could provide a more complete understanding of how physical exercise is embedded in students' broader psychological, educational, and social lives.

## 5 Conclusion

The present study examined the association between physical exercise volume and psychological flourishing among Chinese college students by integrating variable-centered and person-centered perspectives. The findings showed that higher physical exercise volume was positively associated with psychological flourishing, and that this association included significant indirect associations via self-compassion, body appreciation, and their serial association. The latent profile analysis further identified four distinct positive self–body relationship profiles, indicating meaningful heterogeneity in how self-compassion dimensions and body appreciation were configured among students. Physical exercise volume was associated with profile membership, and psychological flourishing differed across profiles, with students in the high self-compassion–high body appreciation profile reporting the highest level of flourishing and those in the low self-compassion–low body appreciation profile reporting the lowest. Overall, these findings suggest that the relationship between physical exercise and flourishing among college students is closely connected with students' ways of relating to themselves and their bodies, and that both self-compassion and body appreciation are important psychological resources to consider in university-based positive mental health promotion. Given the cross-sectional nature of the data, these findings should be interpreted as theoretically meaningful associations that require confirmation in longitudinal and intervention studies.

## Data Availability

The de-identified original data supporting the conclusions of this article are included in the article/Supplementary material as Supplementary Data Sheet 1. Further inquiries can be directed to the corresponding author.
